# Accuracy of different tooth surfaces on 3D printed dental models: orthodontic perspective

**DOI:** 10.1186/s12903-020-01338-6

**Published:** 2020-11-25

**Authors:** Ting Dong, Xiaoting Wang, Lunguo Xia, Lingjun Yuan, Niansong Ye, Bing Fang

**Affiliations:** 1 Department of Orthodontics, Ninth People’s Hospital Affiliated to Shanghai Jiao Tong University, School of Medicine, No. 639 Zhizaoju Road, Shanghai, China; 2 Shanghai Key Laboratory of Stomatology & Shanghai Research Institute of Stomatology, National Clinical Research Center of Stomatology, Shanghai, China

**Keywords:** Printing, Three dimensional, Dental models, Tooth surfaces

## Abstract

**Background:**

Few studies have been reported regarding the accuracy of 3D-printed models for orthodontic applications. The aim of this study was to assess the accuracy of 3D-printed dental models of different tooth surfaces.

**Methods:**

Thirty volunteers were recruited from the hospital, and then their dental models were produced by means of oral scanning and a stereolithography-based 3D printer. Each printed model was digitally scanned and compared with the oral-scanned STL file via superimposition analysis. A color map was used to assess the accuracy of different surfaces (occlusal, buccal, lingual) of anterior and posterior teeth. The Tukey test was used to evaluate the differences between the superimposition.

**Results:**

Statistically significant differences were found in the average deviations of different tooth surfaces (*P* < 0.05). The mean average absolute deviations of the occlusal surfaces of posterior teeth were greater than those of other surfaces. Percentages of points beyond the upper and lower limits of different tooth surfaces displayed the same results (*P* < 0.05).

**Conclusions:**

Occlusal surfaces, especially pits and fissures of posterior teeth on 3D printed maxillary dental models, showed greater distortions than those of other teeth and regions.

## Background

Rapid prototyping (RP) technologies provide the possibility for a physical dental model to be generated from digital data economically and conveniently. These three-dimensionally printed dental models have saved dentists from the dilemma of storage space, risks of damage, and the inconvenience of miscommunication. The 3D-printed dental model is increasingly used in orthodontic diagnosis and treatment. However, it is prone to deviations due to the accuracy of the 3D printer.

Currently, the most commonly used techniques for orthodontics applications are PolyJet and stereolithography (SLA). Due to its high printing resolution and fast forming speed, there has been an increase in the number and availability of SLA-based printers in recent years [1]. Digital light processing (DLP) is a subset technology of SLA printing that uses a projected planar image of light to the photopolymerized resin [2]. During this process, the printing plate moves upward along the Z-axis at a given distance (for example, 0.025 mm, 0.05 mm, 0.1 mm), and photopolymer resin is projected to the light of a specific wavelength (405 nm) that cures a cross-section layer by layer. This printing process is repeated until the model is complete. It usually takes up to several hours for a dental model to be printed with this technology, depending on the printer’s Z-axis print layer thickness [3].

However, little research has been reported regarding the accuracy of 3D-printed models for orthodontic applications. Most of the literature compared linear measurements of 3D-printed models with those of stone models [4–7]. Zhang et al. compared the accuracies of 3D-printed dental models using three types of DLP and SLA printers at different thicknesses and found that the printing accuracy was higher at 50 μm of all the printers [8]. Park and Shin [9] compared the accuracy and reproducibility of conventional dental casts and 3D-printed models fabricated by three types of printers. The results showed that the conventional method’s volumetric changes in casts were significantly smaller than those of 3D-printed casts. Brown et al. [10] compared the tooth and arch measurements of three model types (digital, DLP, and PolyJet) with stone models. Their results indicated high degrees of agreement among all types of models for all measurements, except the crown height measurements between the stone and DLP models. Linear measurements on printed models were found to be slightly less accurate compared to the same measurements performed on plaster models (in a range between 0.20 and 0.30 mm) [5, 11]. However, this difference was in the range of clinical acceptance and similar to the reliability error determined for manual measurements [12, 13]. As a consequence, prototyped models are considered accurate enough for orthodontic study models. More and more orthodontic appliances are being manufactured based on 3D-printed models. The surface suitability of the model will directly affect the production and intra-oral placement of the appliances. Therefore, the accuracy of tooth surfaces is critical. Models with inaccurate 3D-printed surfaces may lead to the poor fit of an orthodontics appliance. The purpose of this study was to assess the accuracy of 3D-printed dental models of different tooth surfaces.

## Methods

A sample of 30 maxillary dental models was included in this study. Thirty volunteers who met the criterion for inclusion (having complete permanent dentition) were recruited from the hospital. Those who met the exclusion criteria (having obvious dental anomalies in size and shape, severe dental crown defects, and severe crowding or rotation) were excluded from the study. First, digital dentition data were obtained by intraoral scan (iTero, Align Technology Inc., San Jose, CA). These scanned data were set as prime STL reference models. Thirty STL reference models were printed with DLP rapid prototyping technologies (DentLab One, SHINING 3D, Hangzhou, China), with a build layer thickness of 0.05 mm [14]. The 3D-printed models were then scanned with the same intraoral scan as the STL test models.

**Fig. 1:**
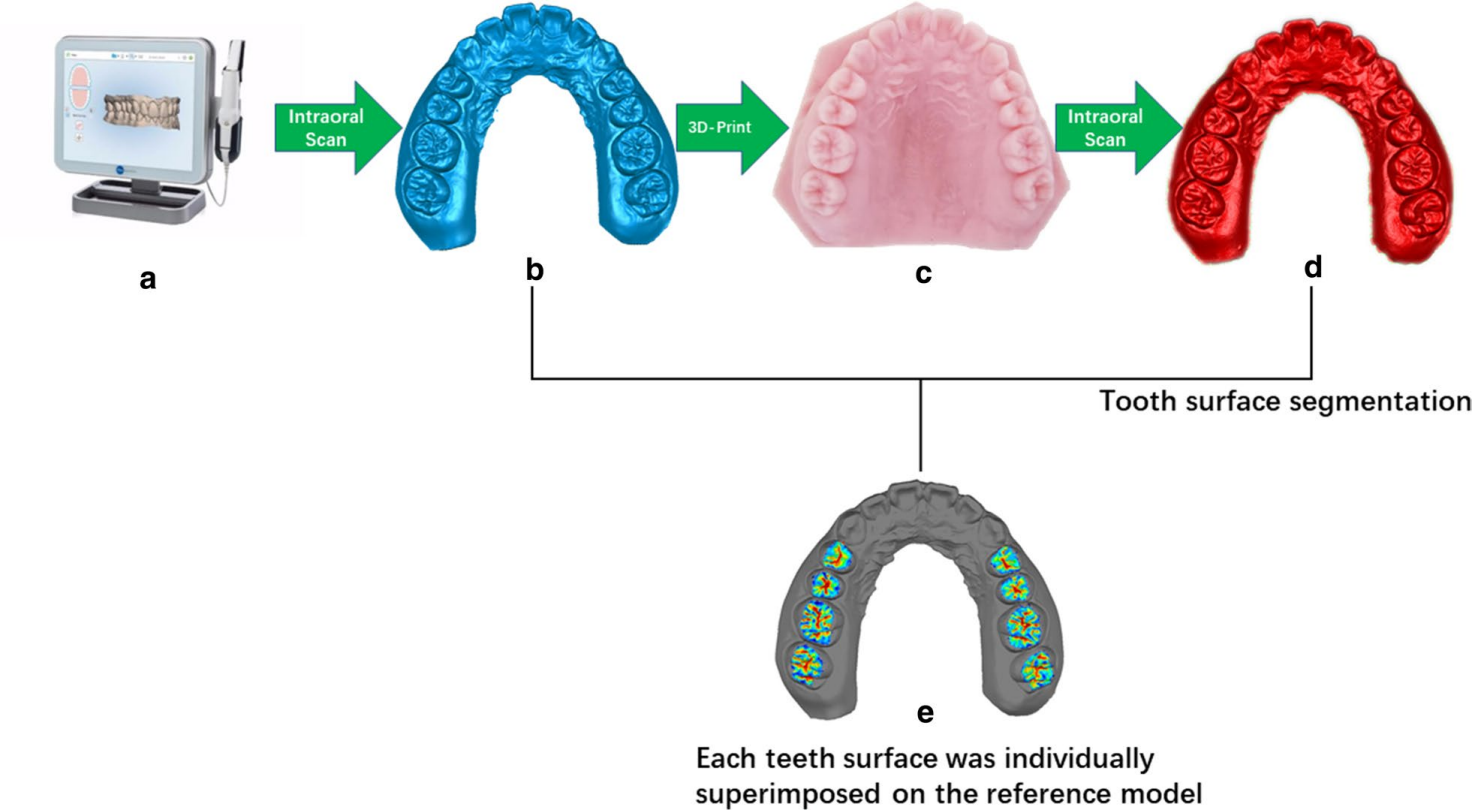
The schematic figure, illustrating the design of this study. Scanning models generated from intraoral scans and 3D-printed models were exported to Geomagic Control software. Tooth surface segmentation was performed on 3D-printed scanning models, and each tooth surface was individually superimposed on the reference model

**Fig. 2:**
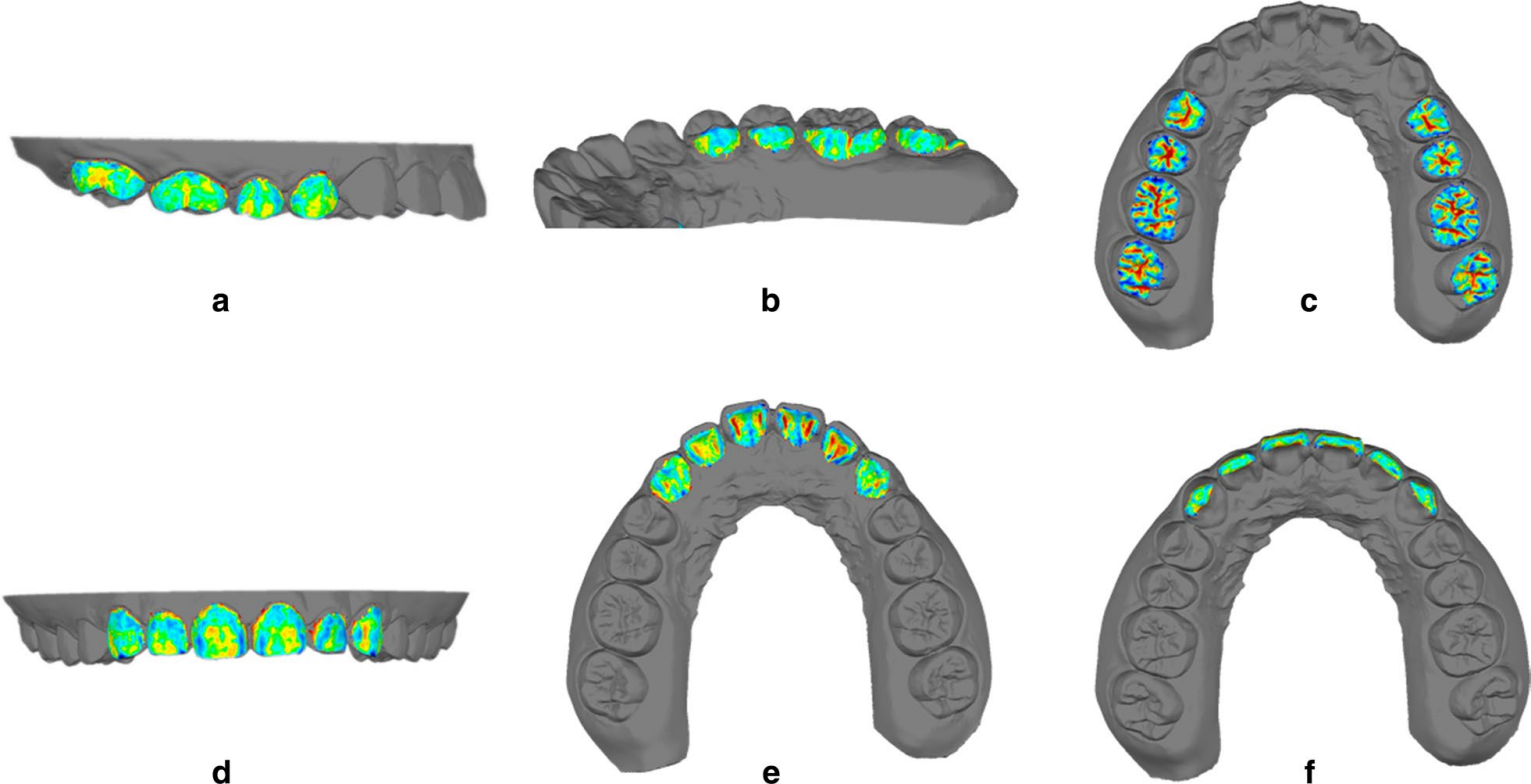
The color map shows the differences in different tooth surfaces between the STL reference model and the STL test model. Each tooth surface was individually superimposed on the reference model. **a** Buccal surfaces of posterior teeth (BP). **b** Lingual surfaces of posterior teeth (LP). **c** Occlusal surfaces of posterior teeth (OP). **d** Buccal surfaces of anterior teeth (BA). **e** Lingual surfaces of anterior teeth (LA). **f** Occlusal surfaces of anterior teeth (OA). The darker the color, the larger the variation; the lighter the color, the smaller the variation. Red and blue showed greater deviations than yellow and green

The digital models generated from both intraoral scan and 3D-printed models were exported to Geomagic Control software (3D Systems, Rock Hill, SC) as STL format files for model superimposition. To standardize the segmentation, the gingival margin was set as a gingival boundary, 1 mm from the incisional margin as an incisal boundary, and marginal ridge as a mesiodistal boundary for anterior teeth. The gingival margin was set as a gingival boundary, marginal ridge as an occlusal and mesiodistal boundary for posterior teeth. Each tooth of the 3D-printed model was segmented separately into buccal, lingual, and occlusal surfaces and superimposed with the corresponding tooth of the STL reference model, then merged to calculate the deviation of the STL reference model (Fig. 1). With ‘canine to canine’ as the anterior region and ‘first premolar to second molar’ as the posterior region, the superimposition was done separately for the buccal surfaces of anterior teeth (BA), lingual surfaces of anterior teeth (LA), occlusal surfaces of anterior teeth (OA), buccal surfaces of posterior teeth (BP), lingual surfaces of anterior teeth (LP), and occlusal surfaces of anterior teeth (OP) (Fig. 2). The segmenting procedure was done twice independently by two researchers at an interval of 1 day. Observer consistency was calculated for reliability. Geomagic software showed the means and standard deviations of different tooth surface distances between the STL reference models and the STL test models. A 0.10-mm threshold parameter was set as the critical value for analyzing deviations between the STL reference model and the STL test model [15]. Any points in the test file deviating from the reference file by more than 0.10 mm in the positive or negative direction were considered to be beyond the upper or lower limits, accordingly. Reports were generated for separate calculation of the total positive and negative deviations.

IBM SPSS Statistics 20.0 (IBM, Chicago, IL) was used for statistical analysis. The Tukey test was used to evaluate the differences between the superimposition of the STL test model and the STL reference model. *P* values less than 0.05 were considered to be significant.

## Results

The repeatability of the measurements in this study was high, as the intraclass correlation coefficient value above 0.8. The color map showed the deviations of the STL test model and the STL reference model (Fig. 2). A 0.10-mm threshold parameter was set as the critical value for analyzing deviations between the reference file and each test file. The darker the color, the larger the variance, and the lighter the color, the smaller the variance, i.e., red and blue, showed greater deviations than yellow and green. It could be seen from the color map that the colors of occlusal surfaces of posterior teeth were much darker than those of other surfaces.

**Fig. 3:**
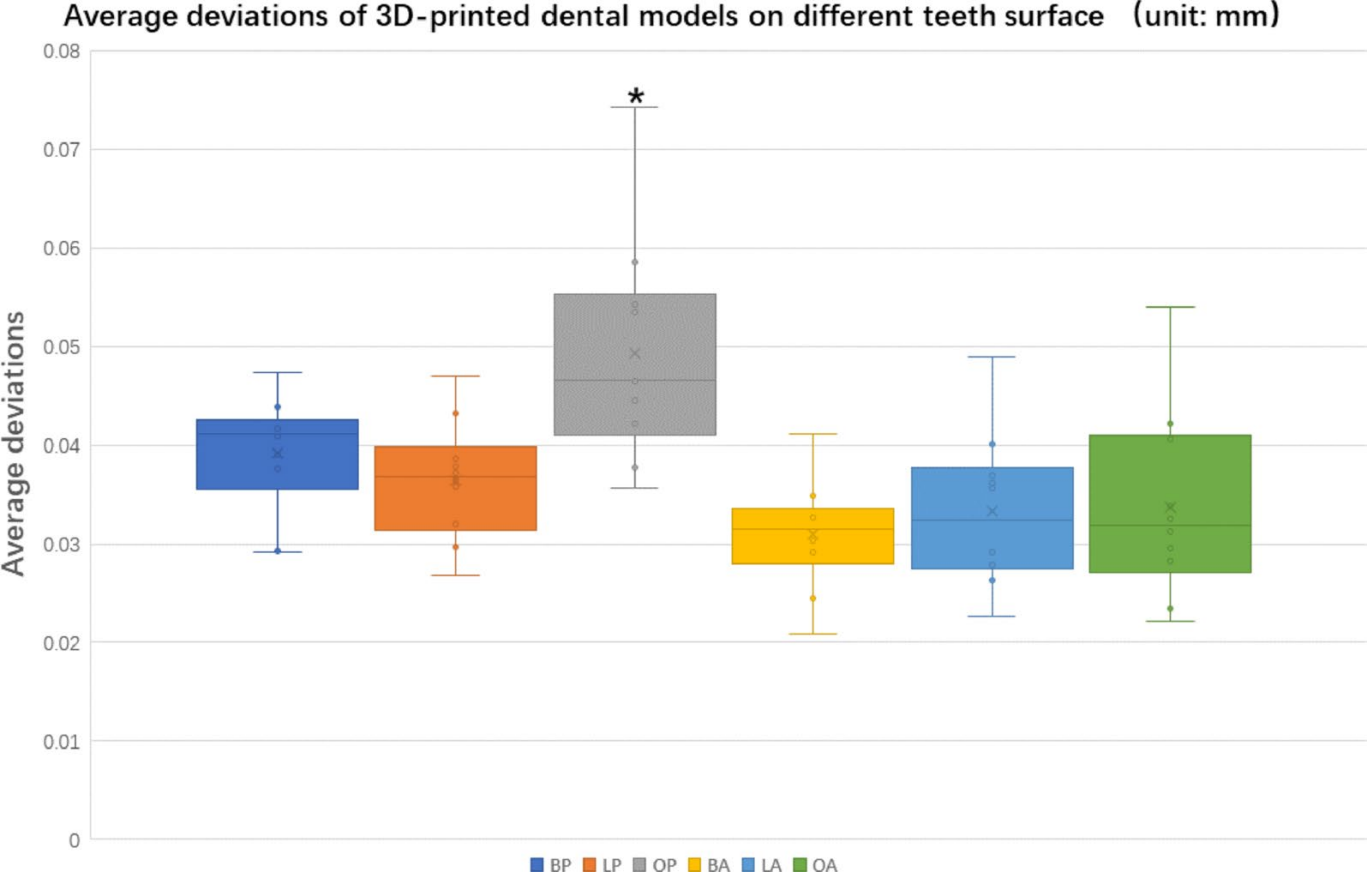
Average deviations of 3D-printed dental models on different tooth surfaces. The average deviations of the occlusal surfaces of posterior teeth were significantly greater than those of other surfaces

The average deviations of 3D-printed dental models of different tooth surfaces were then collected and compared. The average deviation of the anterior region was generally smaller than that of the posterior region, which can be seen clearly from the box chart (Fig. 3). The means and standard deviations of the occlusal surfaces of posterior teeth were greater than those of other surfaces, with a statistically significant difference (*P* < 0.05).

**Fig. 4:**
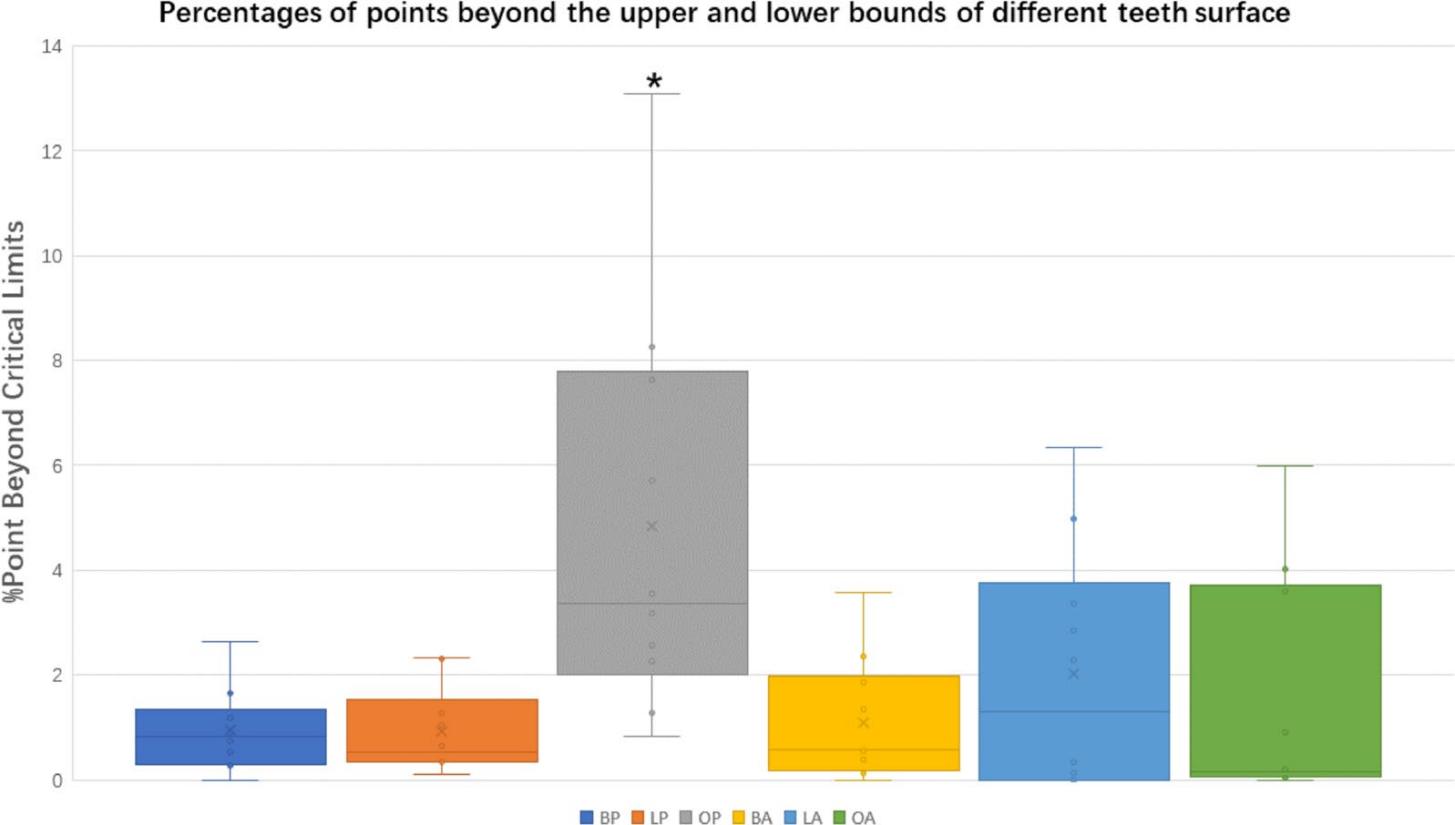
Percentages of points beyond the upper and lower limits of different tooth surfaces. A 0.10-mm threshold parameter was set as the critical value for analyzing deviations between the plastic and the 3D-printed models. The occlusal surfaces of posterior teeth showed significantly higher percentages of points beyond the upper and lower limits

Percentages of points beyond the upper and lower limits of different tooth surfaces were also compared. The variance between the different tooth surfaces was more pronounced. The occlusal surfaces of posterior teeth appeared to have higher percentages of points beyond the upper and lower limits, which showed greater deviation from the reference models (*P* < 0.05) (Fig. 4).

## Discussion

Few investigations have focused on deviations of the different tooth regions (anterior and posterior teeth) and different tooth surfaces. This study compared deviations of 3D-printed models and reference models on buccal, lingual, and occlusal surfaces of anterior and posterior teeth. It could be seen that the average deviation of the posterior dentition of the 3D-printed model was more obvious than that of the anterior region. Kim et al. [16] evaluated four types of 3D-printed models (SLA, DLP, FFF, and PolyJet techniques) for tooth, arch, and occlusal measurements. They found that the difference was larger in the posterior region than in the anterior region in superimposed 3D digital models, which is consistent with the results of this study.

The results of our research demonstrated that the occlusal surfaces of posterior teeth of 3D-printed models deviated primarily from those of the intraoral scan model. The average absolute deviations and percentages of points beyond the upper and lower limits of different tooth surfaces provided confirmatory evidence for this discovery. Although in this study, the average difference between the 3D-printed model and the reference model appeared as a small inclusion occlusal surface, the deviation in the region of posterior pits and fissures was obvious. In this region, the dark red color showed the deviation here to be even more than 0.1 mm, which may have some significant clinical effects. For example, a 3D-printed template or tray cannot be fitted well onto this 3D-printed working model in the laboratory.

**Fig. 5:**
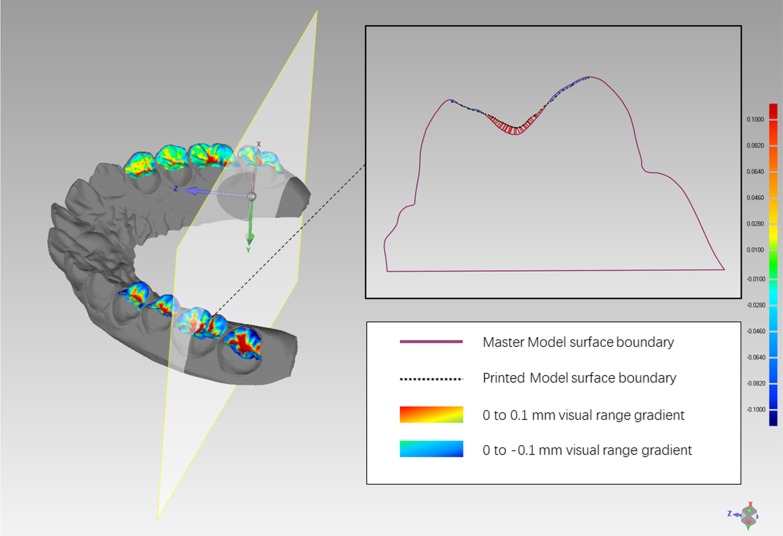
Cross-section of the superimposition. From a cross-section of the superimposed files, it can be seen that there is an obvious difference between the surfaces of the STL reference model and the STL test model, especially in the groove region of the OP

Printing errors in the 3D-printed model can arise from each link of the printing process and the parameters thereof. These include residual polymerization of the resin, effects of support structures, print resolution (X and Y planes), layer thickness (Z plane), and surface finishing [17]. Favero et al. investigated the effect of print layer height on the accuracy of 3D-printed models using three-layer heights (25, 50, and 100 μm) and found that the 25-μm and 100-μm layer height groups had the greatest and least deviations, respectively [1]. Keating et al. examined one SLA model and found statistically significant differences in the Z plane compared with its corresponding stone model and hypothesized that it might be due to the greater layer thickness of the investigated SLA model (0.15 mm) [18]. The relatively significant deviations found in this research may result from the complex morphology of the occlusal surfaces of posterior teeth. Buccal and lingual surfaces are relatively flat and smooth compared with occlusal surfaces, whilst the morphology of the occlusal surfaces of posterior teeth is hilly, particularly in deep pits and fissures. During the rapid prototyping process, the photosensitive resin, which is sticky and requires manual cleaning, will be cured by the ultraviolet laser [19]. The liquid adhesive can flow along the smooth surface but can easily remain on the pits and fissures of the occlusal surfaces of posterior teeth. If it is not cleaned completely or not cleaned in time, the material at the bottom of the groove will cure itself, as occurs during pit and fissure sealing. The deeper the fissure, the greater the deviation (Fig. 5). To solve this problem, technicians can fill the deep grooves in the stone model or digital model in advance to minimize the 3D-printing error.

For the DLP system, only one type of printer was used in this research, which may have been a limitation in our study. Numerous studies have compared the measurements made on 3D-printed models and traditional casts, and different rapid prototyping techniques. Dietrich et al. investigated the accuracy of the SLA and PolyJet systems through surface superimposition [20]. They concluded that the PolyJet models showed greater accuracy than the SLA models, but the precision measurements favored the SLA models. Both systems were suitable for clinical use. Brown et al*.* assessed the accuracy of 3D-printing techniques by tooth and arch measurements and concluded that both the DLP and PolyJet printers were clinically acceptable due to high degrees of agreement between the printed and stone models [10].

The potential errors may also be created by scanners and its procedures. Intraoral scanning and digital models are used widely in the clinic nowadays, such as iTero used in this study. The precision and potential scan errors have been studied by many scholars. Flügge TV et al. [21] evaluated the precision of digital intraoral scanning under clinical conditions. They found that the precision of the intraoral iTero scan is similar to the values documented in the literature with conventional polyether impressions (61.3 ± 17.9 μm) for the reproduction of the intraoral situation. So they concluded that virtual models created with the iTero could be used for treatment planning and manufacturing of tooth-supported appliances. Renne W et al. [22] also evaluated iTero with pretty good precision and trueness. However, the possible errors of the intraoral scan are bigger than the extraoral scan due to patient-relevant elements and complex oral environment [21]. The potential scan errors may have some small effects on creating digital models in this study, but the results were reliable.

However, few investigations have focused on deviations of the different tooth regions (anterior and posterior teeth) and surfaces. This study compared deviations of 3D-printed models and reference models on buccal, lingual, and occlusal surfaces of anterior and posterior teeth. No study has drawn firm and reliable conclusions as to whether the deviations between 3D-printed models and a reference model are clinically acceptable. It remains controversial whether differences in dimensions between the reference model and the 3D-printed models affect the accuracy of orthodontic appliances. Kasparova et al*.* compared traditional plaster casts, digital models, and 3D-printed models and found 3D-printed models to have advantages over traditional plaster casts due to their accuracy and price [6]. Wan Hassan et al. compared the accuracy of measurements made on rapid prototyping and stone models with different degrees of crowding [4]. They found significant differences for all planes in all categories of crowding except for crown height in the moderate crowding group and arch dimensions in the mild and moderate crowding groups. They concluded that the rapid prototyping models were not clinically comparable with conventional stone models.

Intraoral or extraoral scanning is becoming more common and may even replace traditional models in the future. But there is no clear evidence as to whether digital models and 3D-printed models can take the place of stone models to produce some orthodontic appliances in the laboratory. Even designed and produced with digital models, those appliances still need to be tried on the 3D-printed models. Relative to the mandibles, maxillary appliances are more common in the clinic, such as TPA (Trans-Palatal Arch) and expander. Therefore, only maxillary models were used in this research. Further, due to the relatively obvious print errors on the occlusal surfaces of posterior teeth, some appliances and templates made with digital models cannot be fully placed on 3D-printed models [23]. Some measurement differences occurred in these 3D-printed dental models will affect the accuracy of manufactured orthodontic appliances, especially the fit on occlusal surfaces. Deviations in the occlusal template for orthognathic surgery will affect the precision of the surgery. 3D-printing technology is also widely used in the design and manufacture of clear aligners. As is well-known, differences between sequential aligners are only 0.2–0.3 mm, so errors over 0.3 mm may influence the expression of tooth movement. Cole et al*.* examined the accuracy of 3D-printed retainers compared with conventional vacuum-formed and commercially available vacuum-formed retainers [24]. The results showed the least deviation from the original reference models in the conventional vacuum-formed retainers and the greatest deviation in the 3D-printed retainers. However, the deviation was clinically acceptable. Further research is needed to confirm the precision and clinical acceptance of 3D-printed models in orthodontics clinics. Scaled-up research with a large sample size, more printer types, and conditions are needed for further study.

## Conclusions

In this study, 3D-printed dental models were produced with the DLP technique from intra-oral scanning. Comparing the print errors of different surfaces of the anterior and posterior teeth of the 3D-printed model, the average deviation of the posterior dentition was more obvious than that of the anterior region. The occlusal surfaces of posterior teeth displayed greater deviations than other regions, especially in pits and fissures. This deviation should be taken into consideration in the implementation of digital orthodontics. Maybe it still not the time for digital models and 3D-printed models to fully replace the traditional models.

## Data Availability

The datasets used and/or analyzed during the current study are available from the corresponding author on reasonable request.

## Abbreviations

RP: Rapid prototyping
SLA: Stereolithography
DLP: Digital light processing
3D: Three-dimensional
BA: Buccal surfaces of anterior teeth
LA: Lingual surfaces of anterior teeth
OA: Occlusal surfaces of anterior teeth
BP: Buccal surfaces of posterior teeth
LP: Lingual surfaces of anterior teeth
OP: Occlusal surfaces of anterior teeth
